# Artemisinin Derivatives Target Topoisomerase 1 and Cause DNA Damage *in Silico* and *in Vitro*

**DOI:** 10.3389/fphar.2017.00711

**Published:** 2017-10-09

**Authors:** Onat Kadioglu, Ariel Chan, Alena Cong Ling Qiu, Vincent Kam Wai Wong, Vanessa Colligs, Sabine Wecklein, Halima Freund-Henni Rached, Thomas Efferth, Wen-Luan Wendy Hsiao

**Affiliations:** ^1^Department of Pharmaceutical Biology, Institute of Pharmacy and Biochemistry, University of Mainz, Mainz, Germany; ^2^State Key Laboratory of Quality Research in Chinese Medicine, Macau University of Science and Technology, Macau, China

**Keywords:** artemisinin, cancer, DNA damage, molecular docking, topoisomerase

## Abstract

DNA topoisomerases 1 and 2 are enzymes that maintain DNA topology and play important essential genome functions, including DNA replication and transcription. Aberrant topoisomerases cause genome instability and a wide range of diseases, cancer in particular. Both Topo 1 and 2 are the targets of valuable anticancer drugs, such as camptothecin. It has been previously shown that artemisinin, a sesquiterpene lactone from *Artemisia annua* L. also known as *qinghaosu*, possesses anti-cancer effects and one of its derivatives, artesunate inhibits Topo 2. In this study, we evaluated artemisinin and 40 derivatives as potential Topo 1 inhibitors at first by *in silico* molecular docking analyses. Five compounds that showed comparable binding energies and similar docking poses were selected for *in vitro* cytotoxicity test and Comet assay for DNA damage. WWLL-013, WWLL-022, and WWLL-1098 showed the lowest binding energy also induced DNA damage in the Comet assay. CMK-0298 and CMK-0398 intercalated into DNA and induced mild DNA damage. All selected compounds, WWLL-013 in particular, were more cytotoxic toward the rat tumor cells than to the normal cells. In conclusion, the artemisinin derivatives such as CMK-0298, CMK-0398, WWLL-013, WWLL-022, and WWLL-1098 can be further developed as Topo 1 inhibitors.

## Introduction

DNA topoisomerases are classified as type 1 and type 2 (Wang, 2002; Forterre et al., 2007). Type 1 topoisomerases (Topo 1) change the degree of supercoiling of DNA by single-strand breaks and religation, while type 2 topoisomerases (Topo 2) cause double-strand breaks. The enzymes relax supercoiling and torsional tension arisen during transcription and DNA replication, then permit DNA strand passage during early mitosis (Wang, 1985; Brill and Sternglanz, 1988; Kim and Wang, 1989). Six topoisomerase genes were discovered: two Topo 1 genes (TOP1 and TOP1mt), two Topo 2 genes (TOP2α and β), and two topoisomerases 3 (TOP3α and β) (Wang, 2002; Pommier, 2009).

Eukaryotic Topo 1 inserts transient breaks into duplex DNA. It breaks and rejoins one strand of duplex DNA resulting in the relaxation of both positively and negatively supercoiled DNA (Camilloni et al., 1989), whereas Topo 1 from *E. coli* can only relax negatively supercoiled DNA (Tse-Dinh et al., 1983). The relaxed DNA migrates as a series of bands of different sizes rather than a single band, known as topoisomers, which are DNAs of different linking number. This is a measure of a fundamental property of a closed-circular DNA molecule (Shimada and Yamakawa, 1984). Topo 1 catalyzes the equilibrium between these isomers. The energy difference between molecules of similar linking number around the average linking number of relaxed DNA is less than the thermal energy available at normal temperatures. Hence, the equilibrated sample consists of a mixture of topoisomers (Bates and Maxwell, 2005). Migration in an electric field through an agarose gel for circular molecules of the same molecular weight, is essentially a measure of writhe, which describes how the helix axis coils in space. Although adjacent bands differ in linking number, associated differences in writhe, which can cause their different mobility in the gel (Bates and Maxwell, 1997; Bates et al., 2013).

In general, whether a topoisomer of a given linking number is positively or negatively supercoiled or relaxed depends on the conditions, i.e., on the number of helical DNA repeats (Bates and Maxwell, 2005). An important factor influencing the twist and helical repeats of DNA is the presence of intercalating molecules. An intercalator such as ethidium bromide (Neidle and Abraham, 1984) contains a planar, usually polycyclic, aromatic structure, which inserts itself between two base pairs of double-stranded DNA. This causes local unwinding of the DNA helix, resulting in overall increase in the helical repeat that is a decrease of the DNA twist. A decreased twist of closed circular DNA results in increased writhe, causing increased DNA mobility. However, some classes of DNA-binding molecules such as netropsin have opposite effects on the DNA helix, i.e., they increase DNA twist.

Topoisomerases play important roles in DNA replication, transcription, mitotic chromosome formation, DNA recombination, and pre-mRNA splicing (Wang, 2002; Soret et al., 2003). Topo 1 is elevated in malignant tumors, including carcinomas of the colon, prostate, ovary and lung, (Husain et al., 1994; Karachaliou et al., 2013; Romer et al., 2013). High expression of Topo 1 and 2 correlates with poor response to treatment and survival of patients with small cell lung cancer (Karachaliou et al., 2013). Topo 1 expression is also closely associated with tumor metastasis and poor prognosis of patients with colorectal cancer (Romer et al., 2013; Silvestris et al., 2014).

Dysfunction of topoisomerases during cell division causes strand breaks, DNA damage and a collapse of the DNA replication complex, which ultimately leads to apoptosis of growing cells. Previously, Topo 1 stimulated research as the molecular target for cancer therapy owing to the discovery of the chemotherapeutic drug, camptothecin (Hsiang et al., 1985). This is a known Topo 1 inhibitor, and Topo 1 mutations can cause resistance to camptothecin (Chrencik et al., 2004). Therefore, the development of new compounds better targeting this enzyme has high priority.

Artemisinin, a sesquiterpene lactone from *Artemisia annua* L., reveals promising activity toward cancer cells *in vitro* and *in vivo* (Efferth, 2006; Lai et al., 2013; Takatani-Nakase, 2014; Tilaoui et al., 2014). Artesunate, a semisynthetic derivative of artemisinin was previously identified as Topo 2 inhibitor (Youns et al., 2009). In the present study, we investigated the activity of artemisinin derivatives to inhibit Topo 1 by molecular docking. Selected compounds were further evaluated by *in vitro* experiments.

Molecular docking can be applied as a virtual drug screening method based on 3D crystallographic structures of target proteins to predict the protein-ligand interactions. The bioinformatical calculations are based on parameters, which influence attraction and repulsion between target and compound, such as van der Waals interactions, hydrogen bonds, electrostatic interactions and hydrophobic interactions (Morris et al., 2008, 2009). The calculated binding energies and interacting amino acid residues characterize the affinity and the activity of a test compound toward its target. The lower the binding energy, the better the binding is of the compound to the target (Schneidman-Duhovny et al., 2004; Mihasan, 2012).

## Materials and methods

### Cell lines and culture conditions

All experiments were carried out on normal and transformed Rat 6 cells. The normal Rat 6 (R6) is an embryonal fibroblast cell line subcloned from an immortalized F2408 rat embryo cell line (Freeman et al., 1970). It has been a useful immortalized normal cell line in cancer research. Transformed Rat 6 (R6T24) cells are derived from the normal Rat 6 cell line transfected with the plasmid pT24, which carries the *c-H-ras* oncogene. Rat 6 and R6T24 cell lines were employed in this study to test the differential responses of normal and transformed cells to the test compounds. The culture medium used was Dulbecco's Modified Eagle's Medium (low glucose formulation) (DMEM; Gibco BRL, USA) supplemented with Basal Medium Eagle amino acids (BME; Sigma-Aldrich, Taufkirchen, Germany), 10% bovine calf serum (CS; Hyclone, USA) plus 5 mg/ml of each streptomycin and penicillin. Cell cultures were maintained in a humidified incubator at 37°C with 5% CO_2_. Cells were subcultured by trypsinizing the cells with PBS containing 0.05% trypsin and 1 mM EDTA and replated at the appropriate seeding cell density per tissue culture (TC) plate (Corning, USA). Culture medium was changed twice a week.

### Chemicals and reagents

Agarose (normal melting and low melting) was purchased from FMC (Houston, USA). Boric acid was purchased from BDH Chemicals (Dawsonville, GA, USA). Dimethyl sulfoxide, ethylenediaminetetraacetic acid-disodium salt, ethidium bromide, trizma base, triton-X-100, cis-platinum, propidium iodide, ribonuclease A, and paraformaldehyde were purchased from Sigma. Ethanol, hydrochloric acid, sodium hydroxide are purchased from Honeywell-Riedel-de Haën (Seelze, Germany). Sodium chloride, topoisomerase and trypsin were purchased from Gibco BRL (USA). Sodium sarcosinate was purchased from Fluka Biochemika (Germany).

### Molecular docking

The protocol for molecular docking was previously reported by us (Zeino et al., 2014). An X-ray crystallography-based 3D structure of wild-type human Topo 1 (PDB ID: 1K4T) and its N722S mutant (PDB ID: 1RRJ) were obtained from Protein Data Bank (http://www.rcsb.org/pdb). This mutant confers camptothecin resistance (Chrencik et al., 2004). Both structures possess a phosphorylated tyrosine at position 723. The selected residues for defined docking were obtained from the docking pose of topotecan (a camptothecin derivative) on human Topo 1 and its interacting residues (Staker et al., 2002), i.e., DA5, DG6, DA7, DC8, DT9, DC117, DT118, DT119, Arg364, Lys532, Asp533, Asn722, Tyr723.

Chemical structures of artemisinin derivatives were retrieved from Pubchem database and the 3D structures were created by Corina Online. Chemical properties and classification of the derivatives were described in the Table S1. The known Topo 1 inhibitors; camptothecin (Baikar and Malpathak, 2010) and topotecan (Kollmannsberger et al., 1999) as well as hydrolyzed topotecan were used as control drugs. A grid box was then constructed to define docking spaces according to the selected residues for defined docking, whereas the whole protein surface was covered for blind docking. Docking parameters were set to 250 runs and 2,500,000 energy evaluations for defined docking, 25,000,000 for blind docking. Docking was performed three times independently by Autodock4 implemented on AutodockTools-1.5.7rc1 (Morris et al., 2009) using the Lamarckian Genetic Algorithm. Corresponding lowest binding energies and predicted inhibition constants were represented as mean ± SD. Visual Molecular Dynamics (VMD) was used to depict the docking poses of artemisinin derivatives and control drugs.

### DNA decatenation analysis

DNA topoisomerases are enzymes that unwind supercoiled DNA. Topo 1 was used to determine, whether the test compounds intercalate into DNA. The test compounds were incubated with closed circular pUC18 DNA in the presence of Topo 1 and electrophoresis was performed in an agarose gel. A competition in the direction of the writhe may result from the simultaneous reaction of the DNA with both Topo 1 and test compound. Two opposite forces appear on DNA during this reaction. Topo 1 relaxes the closed circular DNA decreasing the writhe to zero. On the other hand, if the test compound intercalates into DNA, the writhe increases due to decreased DNA twist. Depending on the strength of the pull from each side, DNA has different conformational states and its mobility shifts.

The plasmid pUC18 (2.69 Kb) was extracted from DH5α, an *E. coli* bacterial strain using Qiagen Spin column and was used in all experiments. The reaction mix contained 50 ng of pUC18 plasmid DNA (1 μl), 2 μl of 5 × reaction buffer (50 mM Tris HCl pH 7.5, 50 mM KCl, 10 mM MgCl_2_, 0.5 mM DTT, 0.1 mM EDTA and 30 μg/ml BSA), two units of Topo 1 enzyme, 1 μl of the test chemical and dH_2_O to make up a final volume of 10 μl. The reaction mix was incubated at 37°C water bath for 2 h and 2 μl of 6 × loading dye was added. The entire volume of 12 μl was then loaded onto a 1% agarose gel without ethidium bromide (EtBr) and electrophoresed in TAE buffer (40 mM Tris- acetate, 1 mM EDTA). The gel was then stained with 50 μg/ml of EtBr for 20 min and viewed with Digital Imaging System (BIO-RAD, USA).

### Comet assay

Cells were treated as described below under “Cytotoxicity Test.” After 6 days treatment, cells were counted and resuspended in an appropriate volume of PBS to reach a final density of ~3,000–10,000 cells/μl. Cell suspensions were kept on ice prior to the comet assay. We applied the same treatment duration to the cell cultures as the duration used for MTT assay.

Fisher's fully frosted microscope slides (Fisher Scientific, UK) were each covered with 100 μl of 0.5% normal melting agarose (NMA) in PBS at 45°C, immediately covered with a coverslip and then kept at 4°C for 10 min to allow the agarose to solidify. Treated or untreated control cells suspended in 10 μl PBS were mixed with 75 μl 0.5% low melting point agarose (LMA) at 37°C. After gently removing the coverslip, the cell suspension was rapidly pipetted onto the first layer of agarose, spread using a coverslip and maintained at 4°C for 10 min. After removal of the coverslip, a third layer of 0.5% LMA (80 μl) at 37°C was added and spread using a coverslip and allowed to solidify at 4°C (this layer was optional). After the removal of the coverslip, the slides were immersed in freshly prepared cold lysing solution (2.5 M NaCl, 100 mM Na_2_EDTA, 10 mM Tris; pH10, 1% sodium sarcosinate with 1% Triton X-100 and 10% DMSO) for at least 1 h at 4°C. The slides were then removed from the lysing solution, drained and placed in a horizontal gel electrophoresis tank side by side. The tank was filled with fresh electrophoresis buffer (1 mM Na_2_EDTA and 300 mM NaOH) to a level slightly above the slides. The slides were left in this buffer for 20 min to allow unwinding of the DNA before electrophoresis. Electrophoresis was conducted at room temperature for 20 min at 25 V, adjusted to 300 mA by raising or lowering the buffer level in the tank. After electrophoresis, the slides were washed gently to remove alkali and detergents which would interfere with ethidium bromide staining, then placing on a tray and flooding them slowly with three changes of 0.4 M Tris pH7.5, each for 5 min. After neutralization, the slides were stained with 50–100 μl of 2 μg/ml ethidium bromide in distilled water, covered with a coverslip, and stored in the dark. Observations were made using Zeiss Axiophot fluorescence microscope (Zeiss, Jena, Germany). Photographs of single cells were taken at 400 × magnification using P1600 Ektachrome film (Kodak, Stuttgart, Germany).

### MTT cytotoxicity assay

The lead compound artemisinin and its derivatives were initially tested for their cytotoxicity effects on Rat 6 and R6T24 cell lines. The toxicity effects of the chemicals were assessed using MTT (3-[4,5-dimethylthiazol-2-yl]-2,5-diphenyltetrazolium bromide) assay at the end of the treatment periods. For the assay, 800 cells were seeded in each well of a 96-well plate in a final volume of 100 μl of DMEM medium supplemented with 10% calf serum. Six duplicate wells were used for each sample. On the next day, cells were fed with drug-containing medium and kept incubated for another 3 or 6 days. Drugs were freshly added to the cultures when the cells were fed every other day with fresh medium. The final concentration of each compound ranged from 0 to 100 μM. DMSO or tetrahydrofuran was used as solvent. Depending on the solubility of the compounds, 1,000 × stock (i.e., 100 mM) of the compounds was prepared. Due to the limitation of the solubility of the test compounds, the maximum test concentrations varied from 25 to 100 μM as designated in the experiment. At the end of the drug treatment, 50 μl of the MTT labeling reagent was added at a final concentration of 0.5 mg/ml to each well. The plate was re-incubated for 4 h in a humidified atmosphere prior to the addition of 100 μl of the solubilizing solution (10% SDS in 0.01 M HCl) into each well. The plate was allowed to stand overnight in the incubator to enhance the complete solubilization of the purple formazan crystals. The plate was scanned for measuring the absorbance using a Dynatech MR5000 (Dynatach, USA) at wavelengths between 550 and 600 nm. The data were expressed as the mean ± SEM (*n* = 6) and were analyzed by the Student's *t*-test with a *p* < 0.05 considered to be significant.

### Real-time PCR-based melting DNA shifting assay in the presence of cisplatin and artemisinin

RNA was isolated from HeLa cells using Qiagen RNasy mini kit (Qiagen). Reverse transcription was performed in 20 μl reaction mixture [Transcriptor Universal cDNA Master (Roche)] with 2.5 μg total RNA. The synthesized cDNA was diluted 1:10 before used as the template. β-Actin primers (5″- CTC TTC CAG CCT TCC TTC CT-3″ –F & 5″ -AGC ACT GTG TTG GCG TAC AG- 3″ -R) were used in real-time PCR (qPCR) assay. The 20 μl qPCR reaction mixture contained 2 μl template; 0.3 μl each of 1 mM forward and reverse primers; 10 μl PowerUp SYBR™ Green Master Mix (Thermo Fisher); 2 μl 0.1 mM compound (artemisinin or cisplatin) or DMSO; 5.4 μl ddH2O. The qPCR was conducted as following: an initial 2 min at 50°C, 10 min at 95°C; followed by 40 cycles of 15 s at 95°C and 1 min at 60°C. The last melting curve stage was conducted at 15 s at 95°C, 1 min at 60°C, 15 s at 95°C. The dissociation curve was analyzed using ViiA 7 RUO software (Life technology, Carlsbad, USA) to demonstrate the shift of melting curve due to the binding of the test compound to DNA molecules.

## Results

### Evaluation of 40 artemisinin derivatives as potential topo 1 inhibitors using molecular docking analysis

**Figure 1:**
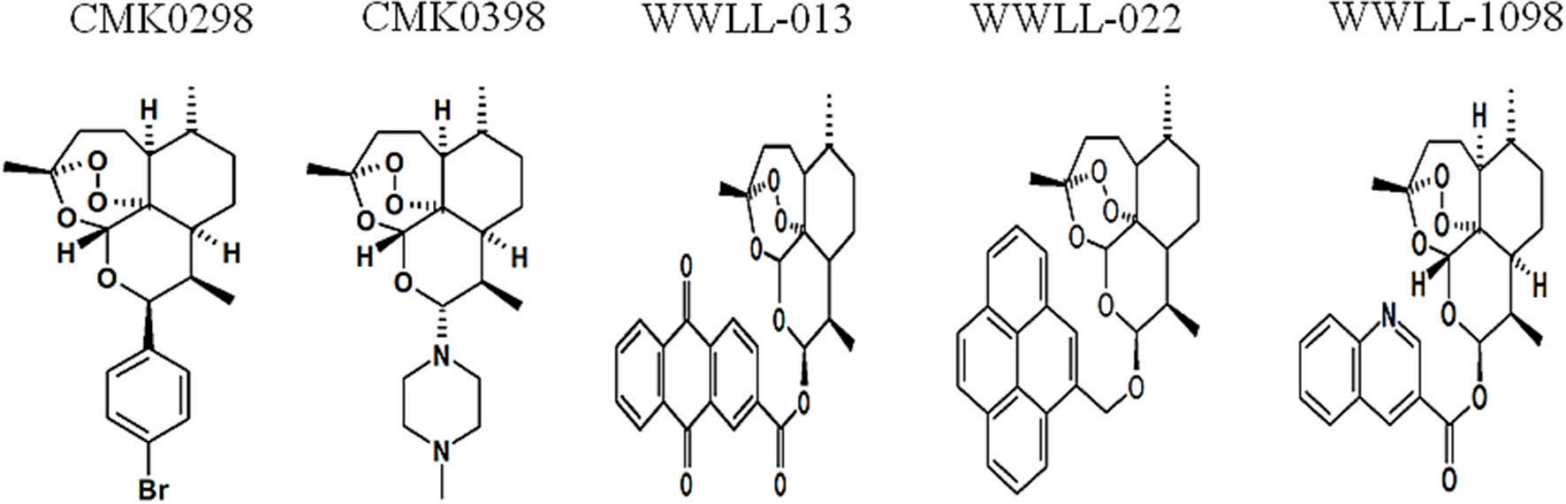
Chemical structures of selected artemisinin derivatives (CMK0298, CMK0398, WWLL-013, WWLL-022, and WWLL-1098).

**Figure 2:**
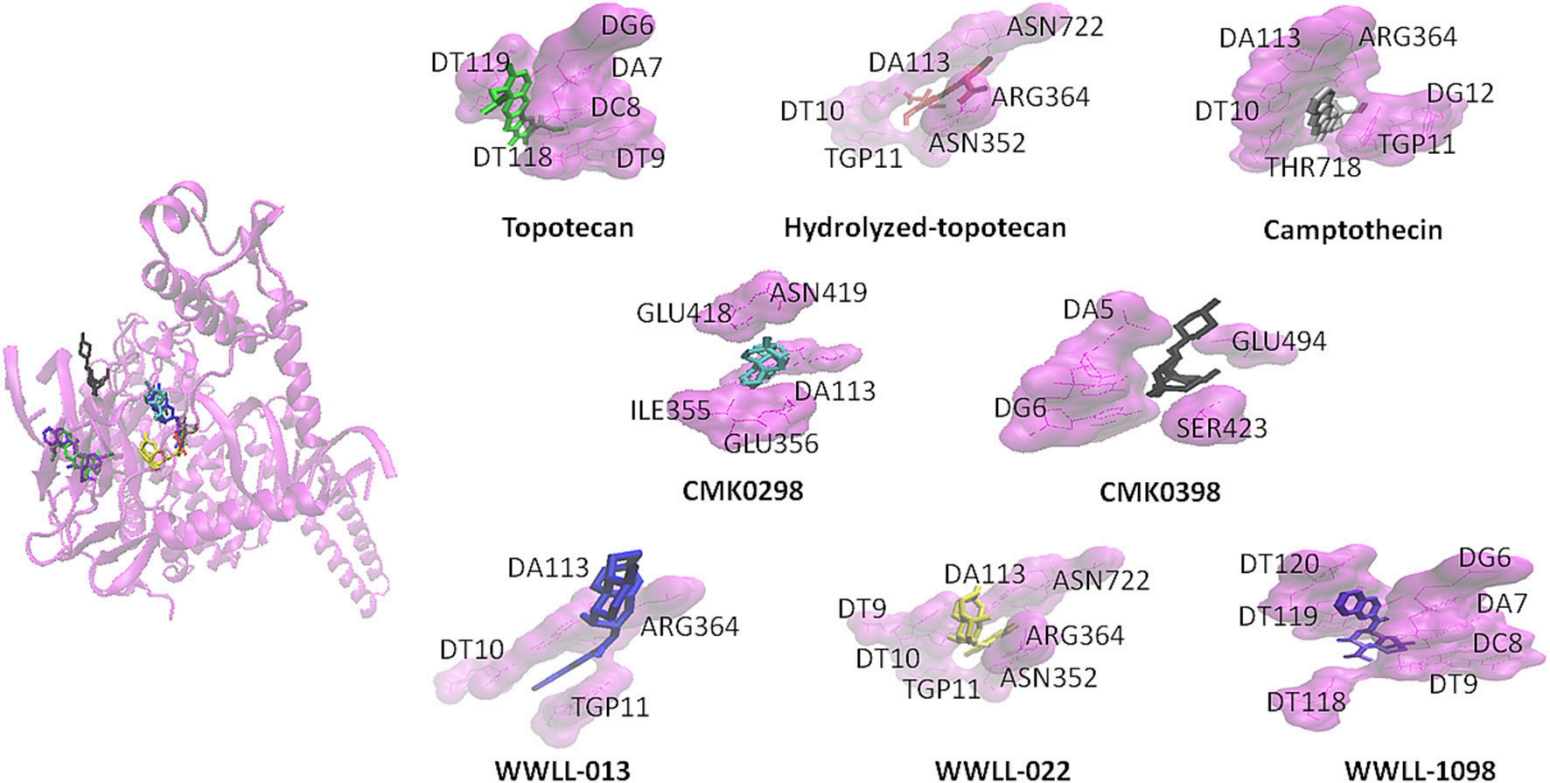
CMK0298 (cyan), CMK0398 (black), WWLL-1098 (violet), WWLL-022 (yellow), WWLL-013 (blue), topotecan (green), hydrolyzed topotecan (red), camptothecin (silver) docking on human Topo 1 (magenta. PDB ID:1K4T).

**Figure 3:**
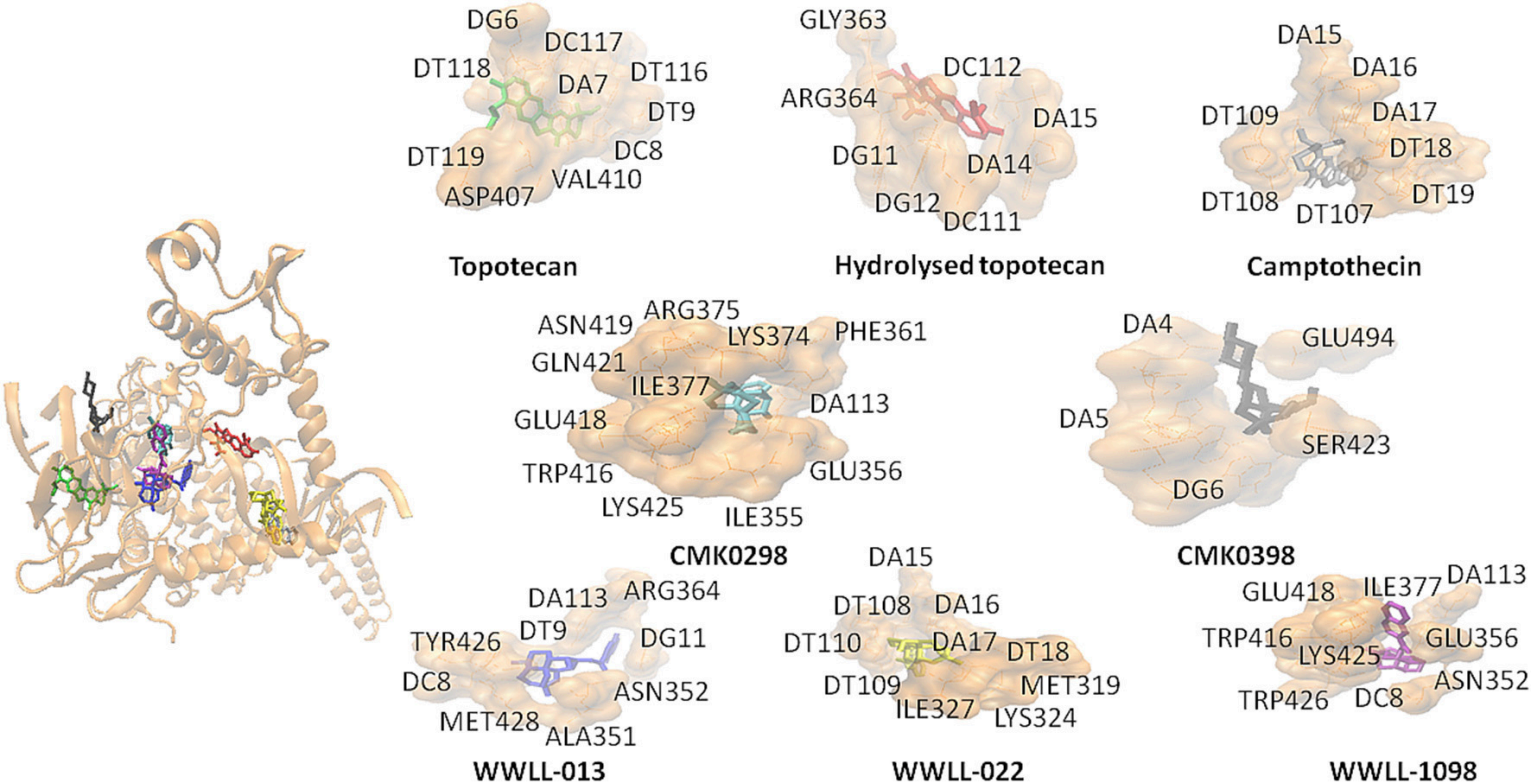
CMK0298 (cyan), CMK0398 (black), WWLL-1098 (violet), WWLL-022 (yellow), WWLL-013 (blue), topotecan (green), hydrolyzed topotecan (red), camptothecin (silver) docking on N722S mutant human Topo1 (orange. PDB ID 1RRJ).

Artemisinin derivatives were firstly evaluated by *in silico* molecular docking on human Topo 1 to identify potential inhibitors. Two protein structures were used: wildtype and N722S mutant Topo 1. Docking poses and binding energies were compared with known inhibitors, i.e., topotecan, hydrolyzed topotecan and camptothecin. The docking results were summarized in Table 1 for docking to the wildtype protein and in Table 2 to the mutant protein. Artemisinin derivatives that showed comparable binding energies and similar docking poses with the control inhibitors were selected for further analyses (they were labeled bold in Tables 1, 2). Chemical structures of the selected artemisinin derivatives (CMK0298, CMK0398, WWLL-013, WWLL-022, and WWLL-1098) are depicted in Figure 1. These selected compounds possessed similar docking poses as the control inhibitors both on wildtype (Figure 2) and mutant Topo 1 (Figure 3).

**Table 1:** Molecular docking analyses of 40 artemisinin derivatives on wild-type human Topo 1 (PDB ID:1K4T).

**Compounds**	**lowest binding energy (kcal/mol)**	**pKi (μM)**	**Interacting residues**	**Residues forming H-bonds**
**WWLL-013**	−12.42	0.001	**DT10, TGP11, DC112, DA113**, GLU208, IL355, **GLU356, ARG364**, LYS374, ILE377, TRP416, GLU418, LYS425	ARG364
**WWLL-022**	−12.21	0.001	DT9, **DT10, TGP11, DA113**, DA114, ALA351, **ASN352, ARG364**, LYS425, TYR426, MET428, GLN442, **ASN722**	DT10, DT9
Topotecan	−12.19	0.001	DA5, DG6, DA7, DC8, DT9, DC117, DT118, DT119	DC117, DT9
WWLL-021	−11.52	0.004	DT10, DG12, DA13, TPG11, DA113, ARG364	DG12
**WWLL-1098**	−10.96	0.009	**DG6, DA7, DC8, DT9, DT118, DT119**, DT120, LYS216	DG6
WWLL-055	−10.79	0.012	DA16, DA17, DT18, DT107, DT108, DT109, DT110, LYS328, ASN331	DA17, DT108, DT109
**CMK0398**	−10.72	0.014	DA4, **DA5, DG6**, SER423, GLU494	Glu494, DG6
Hydrolyzed-topotecan	−10.53	0.019	DT10, TGP11, DC112, DA113, ASN352, GLU356, ARG364, LYS532, ASP533, THR718, ASN722	LYS532
WWLL-0398	−10.45	0.022	DT10, TGP11, DC112, DA113, ASN352, ARG364	DA113
Camptothecin	−10.29	0.029	DT10, TGP11, DC112, DA113, ARG364, ASP533, THR718	ASP533
CAN-28-pt-A	−10.20	0.033	DC8, DT9, DA113, GLU356, LYS374, ILE377, ILE424, LYS425, TYR426	LYS425, TYR426
WWLL-046	−10.19	0.034	DA4, DA5, DG6, DA7, DT119, DT120, DT121	DA4, DA5
WWLL-0998	−10.17	0.035	DC8, DA113, GLU356, ARG375, ILE377, TRP416, LYS425, TYR426	DA113, TRP416
CAN-06	−9.98	0.048	TGP11, DC112, DA113, GLU356, PRO358, LYS374, ILE377, TRP416, GLU418, LYS425	–
**CMK0298**	−9.97	0.049	**DA113**, ILE355, **GLU356**, PHE361, LYS374, ILE377, TRP416, GLU418, ASN419, LYS425	–
WWLL-1198	−9.94	0.052	DT9, DT10, TGP11, DC112, DA113, ASN352, GLU356, ARG364, TRP416, LYS425, TYR426	DA113,DA113, ARG364
2-DEOXY-CAN-06a	−9.62	0.090	DG6, DA7, DC8, DT9, DT116, DC117, DT118, DT119,Lsy 216, Lys409, Lys587	–
WWLL-0598	−9.36	0.139	DA113, DC8, GLU356, TRP416, LYS425, TYR426	DA113, TYR426
WWLL-1598	−9.28	0.157	GLU356, ARG364, LYS374, TRP416, GLU418, LYS425, DC112, DA113, TGP11	GLU356, ARG364
WWLL-049	−9.07	0.226	GLU356, PRO357, LYS374, ARG375, ILE377, TRP416, GLU418, LYS425, TYR426	GLU356, TYR426
AV-003-1	−8.91	0.293	DA113, ILE355, GLU356, PHE361, LYS374, ILE377, GLU418, ASN419, ILE420, GLN421, LYS425	–
WWLL-041b	−8.86	0.319	DG6,DA7, DC8, DT9, DT116, DT118, LYS216	DA7, LYS216
WWLL-0498	−8.86	0.321	DC8, DT9, DA113, LEU220, GLU356, TRP416, ILE424, LYS425, TYR426	TYR426
AV-008-1	−8.69	0.429	DC112, DA113, GLU356, LYS374, TRP416, GLU418, LYS425	TRP416
MW-530	−8.69	0.426	DC8, DA113, ASN352, LYS354, ILE355, GLU356,TRP416, LYS425, TYR426	TYR426
10-Deoxo-artemisinin	−8.64	0.467	DG6, DA7, DC8, DT9, DT10, DC117, DT118, DT119, LYS216	DG6, DA7
AV-007-1	−8.59	0.501	DC112, DA113, ASN352, ILE355, GLU356, LYS374, ILE377, TRP416	–
CMK0498	−8.57	0.520	DC8, DA113, LYS354, ILE355, GLU356, TRP416, ILE424, LYS425, TYR426	LYS425, TYR426
WWLL-076	−8.57	0.522	DA7, DC8, DC117, DT118, DT119, LYS216	LYS216
WWLL-048	−8.45	0.636	DC112, DA113, ILE355, GLU356, LYS374, ILE377, TRP416, GLU418, LYS425	GLU356
WWLL-047b	−8.40	0.694	DA13, DA14, DA15, DA16, DT110, DC111, His266	DC111
AV-004-1	−8.39	0.702	DC112, DA113, GLU356, PHE361, LYS374, ILE377, TRP416, GLU418, ASN419, ILE420, GLN421, LYS425	–
AV-006-1	−8.34	0.767	DC112, DA113, GLU356, PHE361, LYS374, ILE377, TRP416, GLU418, ASN419, ILE420, GLN421, LYS425	–
ARTESUNATE	−8.26	0.875	DG12, DA13,DA14, TGP11, DC112, ARG364	DG12, TGP11
WWLL-071	−8.25	0.902	DA7, DC8, DT10, DC117, DT118, DT119, LYS216	LYS216
WWLL-040b	−8.17	1.030	CG12, TPG11, DC112, DA113, GLY363, ARG364, ASP533, ILE535	TPG11, DG12
2-DEOXY-WWLL040B	−8.11	1.140	GLU356, LYS374, ILE377, TRP416, GLU418, ILE420, LYS425, TYR426	TYR426
WWLL-0698	−8.06	1.240	DC8, DT10, DA113, ASN352, LYS354, ILE355, GLU356, TRP416, LYS425, TYR426	TYR426
AV001-1	−7.60	2.700	IlE355, GLU356, ILE377, TRP416, GLU418, ASN419, LYS425	TRP416
ARTEMETHER	−7.56	3.190	ILE355, GLU356, LYS374, ARG375, ILE377, TRP416, GLU418, LYS425	GLU356, TRP 416
QINGHAOSU	−7.55	2.940	DA113, ILE355, GLU356, PRO357, PRO358, LYS374, ARG375, ILE377, TRP416, LYS425	LYS374
Dihydroartemisinin	−7.22	5.120	DC8, DT9, DA113, ASN352, LYS425, TYR426	DA11, DA113, LYS425, TYR426
2-DEOXY-WWLL-041B	−6.91	8.590	DG12, DA13,DA14, DC111, DC112, DA113, HIS266, ARG364	ARG364
WWLL-053	−6.91	8.560	DA113, ILE355, GLU356, LYS374, ARG375, ILE377, TRP416, GLU418, ASN419, LYS425	–

*Topotecan, hydrolyzed topotecan and camptothecin were used as control and labeled red. The selected compounds and their commonly observed interacting residues with that of topotecan/hydrolyzed topotecan/camptothecin were labeled bold*.

**Table 2:** Molecular docking analyses of 40 artemisinin derivatives on N722S mutant human Topo 1 (PDB ID:1RRJ).

**Compounds**	**lowest binding energy (kcal/mol)**	**pKi (μM)**	**Interacting residues**	**Residues forming H-bonds**
Topotecan	−12.66	0.001	DG6, DA7, DC8, DT9, DT116, DC117, DT118, DT119, ASP407, VAL410	DT9, DC117
**WWLL-013**	−12.58	0.001	**DC8, DT9, DG11**, DA113, ALA351, ASN352, **ARG364**, TYR426, MET428	ARG364, MET428
**WWLL-022**	−11.72	0.003	**DA15, DA16, DA17, DT18, DT108, DT109**, DT110, MET319, LYS324, ILE327	DA17
WWLL-021	−11.09	0.007	DA15, DA16, DA17, DT108, DT109, DT110, MET319, LYS324, ILE327	DA17
WWLL-055	−10.84	0.011	DA16, DA17, DT18, DT107, DT108, DT109, DT110, LYS328, ASN331	DA17, DT108, DT109
**CMK0398**	−10.63	0.016	DA4, DA5, **DG6**, SER423, GLU494	DG6
**WWLL-1098**	−10.56	0.018	**DC8**, DA113, ASN352, GLU356, ILE377, TRP416, GLU418, LYS425, TYR426	TYR426
CAN-06	−10.22	0.032	DG11, DC112, DA113, ILE355, GLU356, PRO357, PRO358, ARG364, LYS374, ILE377, TRP416	–
WWLL-1198	−10.22	0.032	DA113, ASN352, LYS354, ILE355, GLU356, LYS374, ARG375, GLU418, LYS425, TYR426	GLU356, LYS374
WWLL-0998	−10.16	0.036	ASN352, ILE355, GLU356, PRO358, LYS374, ARG375, TRP416, GLU418, TYR426, ILE427	GLU356
CAN-28 pt-A	−10.08	0.041	DG12, DA13, DA15, DT110, DC111	DG12, DA13
WWLL-046	−10.03	0.045	DA4, DA5, DG6, DT120, DT121, DT122,	DA4, DA5
WWLL-0398	−9.96	0.050	DC112, DA113, GLU356, PHE361, ILE377, TRP416, GLU418, LYS425, TYR426	DA113
Hydrolyzed-topotecan	−9.95	0.050	DG11, DG12, DA14, DA15, DC111, DC112,GLY363, ARG364	DG11, DG12, DG12, ARG364
Camptothecin	−9.94	0.052	DA15, DA16, DA17, DT18, DT19, DT107, DT108, DT109	DA16
2-Deoxy-CAN-06a	−9.69	0.078	DA17, DT18, DT107/108/109/110, LYS324, ILE327, LYS328, ASN331	-
WWLL-1598	−9.47	0.086	DT9, DT10, DG11, DG12, DA113, ARG364, ARG488, LYS532, THR718, SER722, LYS751, PTR723	LYS751, DG11
**CMK0298**	−9.37	0.135	DA113, ILE355, GLU356, PHE361, LYS374, ARG375, ILE377, TRP416, GLU418, ASN419, GLN421, LYS425	–
CMK0498	−9.18	0.188	DT10, DG11, DG12, DA113, ARG364, ASP533, ILE535, THR718	DG11
WWLL-0598	−9.09	0.217	DA113, ILE355, GLU356, TRP416, LYS425, TYR426	TYR426
AV-008-1	−9.08	0.220	DA113, GLU356, LYS374, ILE377, TRP416, GLU418	–
AV003-1	−9.07	0.223	DA113, DA114, ILE355, GLU356, PHE361, LYS374, ILE377, GLU418, ASN419, ILE420, GLN421, LYS425	–
WWLL-0498	−9.02	0.244	DT10, DG11, DG12, DA113, ARG364, ASP533, THR718	DG11
AV-007-1	−8.94	0.278	DA113, GLU356, LYS374, ILE377, TRP416, GLU418	–
WWLL-049	−8.83	0.340	DG6, DA7, DC8, DT9, DT118, DT119, LYS216, VAL410, LYS439	DG6, DA7
10-Deoxo-artemisinin	−8.65	0.459	DG6, DA7, DC8, DT9, DC117, DT118, DT119, LYS216	DG6, DA7
WWLL-047b	−8.57	0.523	ILE355, GLU356, LYS374, ILE377, TRP416, GLU418, ASN419, ILE420, LYS425	–
WWLL-48b	−8.55	0.537	DA113, DA114, PHE361, LYS374, ILE377, TRP416, GLU418, ASN419, ILE420, GLN421, LYS425	–
MW-530	−8.54	0.549	DC112, DA113, PHE361, TRP416, GLU418, ILE420, LYS425, TYR426	DA113
WWLL-076	−8.50	0.586	DA113, GLU356, PHE361, LYS374, ILE377, TRP416, GLU418, ASN419, ILE420, GLN421, LYS425	
WWLL-041b	−8.48	0.612	DG12, DA13, DA14, DA15, DT110, DC111, DC112	DA15, DT110, DC111
WWLL-071	−8.40	0.701	DA113, DA114, PHE361, LYS374, ILE377, TRP416, GLU418, ASN419, ILE420, GLN421, LYS425	
AV-004-1	−8.29	0.840	DC112, DA113, DA114, GLU356, PHE361, LYS374, ILE377, TRP416, GLU418, ASN419, ILE420, GLN421, LYS425	–
AV-006-1	−8.20	0.971	DC112, DA113, DA114, GLU356, PHE361, LYS374, ILE377, TRP416, GLU418, ASN419, ILE420, GLN421, LYS425	–
WWLL-040b	−8.11	1.140	DC8, DT9, DA113, GLU356, TRP416, LYS425, TYR426	TYR426
2-DEOXY-WWLL-041B	−7.98	1.420	DC8, DA113, ASN352, LYS354, ILE355, GLU356, TRP416, TYR426, ILE427	TYR426
2-DEOXY-WWLL-040B	−7.97	1.440	DC112, DA113, GLU356, LYS372, ILE377, TRP416, GLU418, LYS425, TYR426	TYR426
ARTESUNATE	−7.83	1.840	DG11, DG12, DA13, DA14, DC112, ARG362, ARG364	DG11, DG11
QINGHAOSU	−7.71	2.220	DA113, GLU356, PRO358, LYS374, ARG375, ILE377, TRP416, LYS425	LYS374
ARTEMETHER	−7.41	3.690	DC8, DT9, DA113, LYS425, TYR426	TYR426
AV-001-1	−7.38	3.930	DA13, DA14, DA15, DT108	DA14, DA15
WWLL-0698	−7.11	6.140	DG11, DG12, DA13, DA14, DC111, DC112, HIS266, TYR268, ARG364	DA14, DC112
WWLL-053	−7.02	6.770	DA13, DA14, DA15, DA16, DT107, DT108, PHE640	DA15
Dihydroartemisinin	−6.94	10.480	DA13, DA14, DA15, DT108, PRO637, LYS638	DA14

*Topotecan, hydrolyzed topotecan and camptothecin were used as control and labeled red. The selected compounds and their commonly observed interacting residues with that of topotecan/hydrolyzed topotecan/camptothecin were labeled bold*.

### The artemisinin derivatives interfered with topo 1 activity as demonstrated by DNA decatenation assay

**Figure 4:**
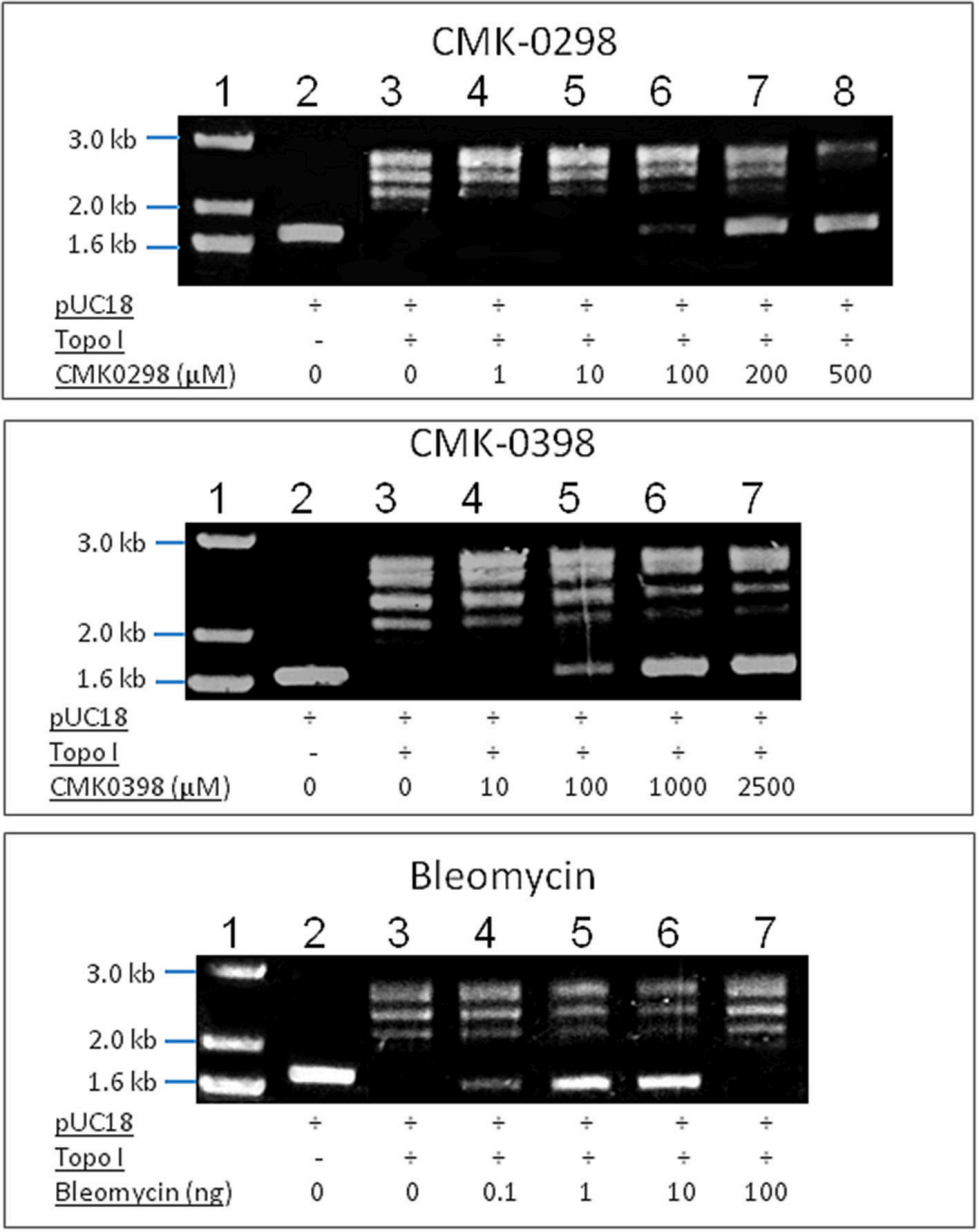
DNA decatenation assay for CMK-0298 and CMK-0398. Bleomycin was used as control compounds. Lane 1, 1 kb DNA marker; Lane 2, supercoiled pUC18 plasmid DNA; Lane 3, Topo 1-treated pUC18.The other lanes show pUC18 DNA treated with Topo 1 plus test compounds at dosages indicated below: CMK-0298: lanes 4–8: 1, 10, 100, 200, 500 μM; CMK-0398: lanes 4–7: 0.01, 0.1, 1, 2.5 mM; Bleomycin: lanes 4–7: 0.1 mg/mL. 10 ng/mL, 1 ng/mL, 0.1 ng/mL; Ethidium bromide: lanes 4–9: 2, 1.5, 1, 0.75, 0.5, 0.2 μg/ml.

To test whether the selected artemisinin derivatives interfere Topo 1 activities, CMK0298 and CMK0398 were incubated with supercoiled pUC18 DNA in the presence of Topo 1 and subjected to agarose electrophoresis. Bleomycin, a cancer therapeutic agent causing DNA strand breaks, was used as positive control for the assay. The force to increase the writhe by intercalating into DNA overrides the force of Topo 1 activity, if the concentration was above 1 μg/ml. Therefore, the DNA isomers were converted to a more compact conformation causing a faster migration in the gel. At saturating amounts of bleomycin (>2 μg/ml), the DNA migrated at similar rates as untreated closed circular DNA (refer to lane 2). At around the equilibrium concentration (point of balance between the opposing forces) or below (0.75 μg/ml), the DNA band pattern was similar to that of treatment only with Topo 1 (refer to lane 3). This indicated that Topo 1 activity was superior to DNA intercalation (if any) in the reaction. The conformation of the topoisomers at these low concentrations appeared not to be affected by treatment of with test compounds. Similar findings were observed with bleomycin. Closed circular pUC18 DNA that was incubated with Topo 1 together with the compounds, CMK-0298 and CMK-0398, which showed similar results as bleomycin (Figure 4). However, a noticeable difference was observed for the equilibrium concentrations of these test compounds, which were higher than those of the classical intercalators. One possible explanation may be the smaller size of these test compounds. Hence, more molecules were required to achieve equivalent unwinding effects compared to those of classical DNA intercalators, such as bleomycin.

### Artemisinin derivatives showed various degree of DNA damage as tested by comet assay

**Figure 5:**
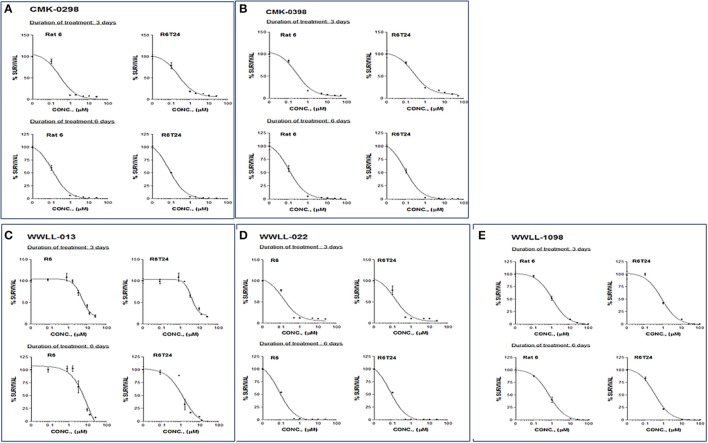
MTT assays of artemisinin derivatives on the normal R6 and transformed R6 (R6T24) cells treated with CMK-0298 **(A)**, CMK-0398 **(B)**, WWLL-013 **(C)**, WWLL-022 **(D)** and WWLL-1098 **(E)** respectively.

**Figure 6:**
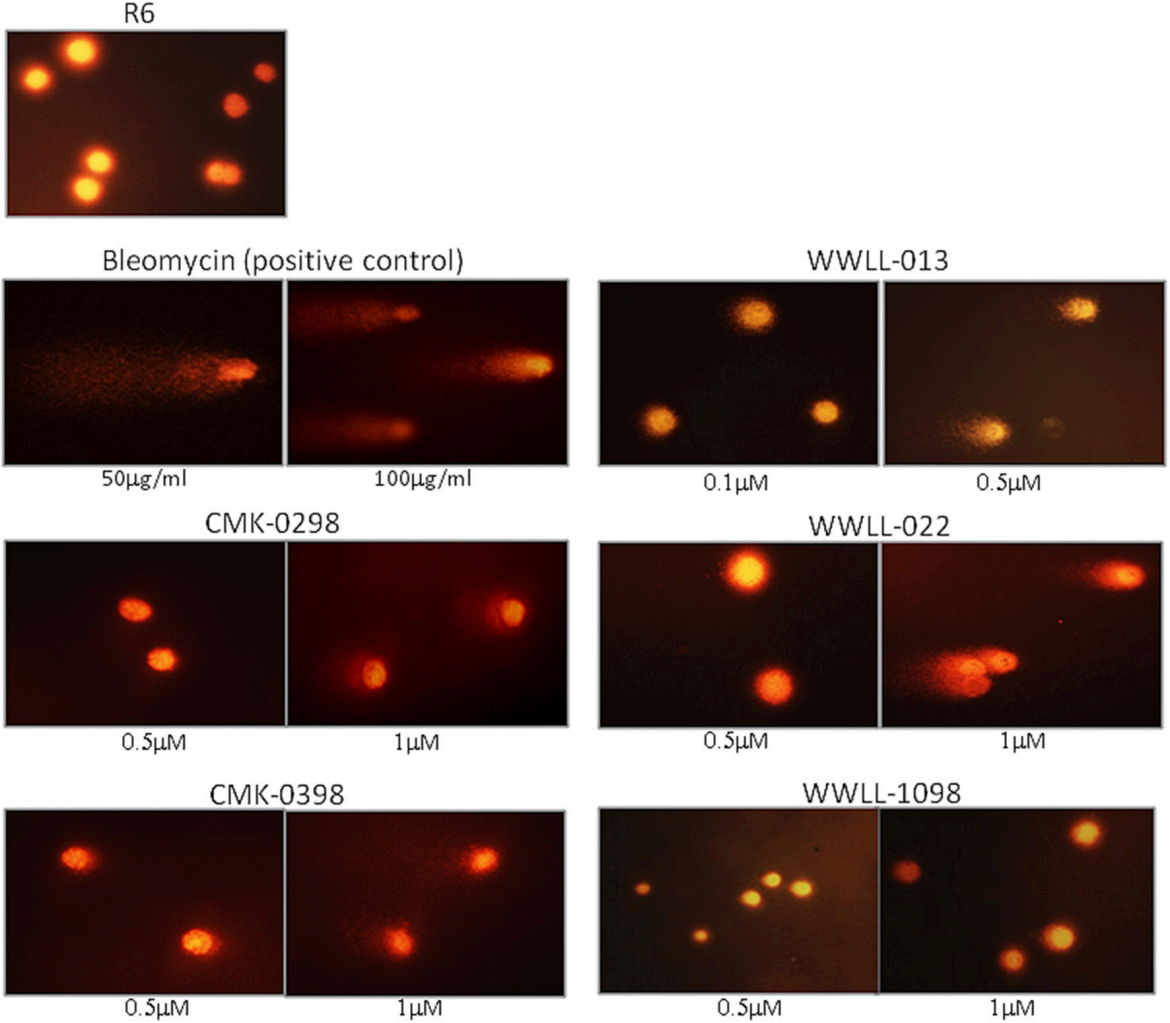
Comet assays of R6 cells treated with CMK-0298, CMK-0398, WWLL-013, and WWLL-022. Bleomycin was used as the control compound.

The dysfunction of Topo I induced by inhibitors is often associated with DNA damage. We then tested the effect of the selected artemisinin derivatives on DNA damage based on the formation of comet tail upon drug treatment. The data showed that CMK-0298, CMK-0398, WWLL-013, WWLL-022, and WWLL-1098 all showed various degree of positivity on Comet assay (Figure 4). The induced dosages ranged from 0.5 to 1 μM, which were in general higher than the IC_50_ values of the tested derivative (Figure 5, Table 3). Bleomycin caused DNA damage at relative concentrations (Figure 6). Since CMK-0298 and CMK-0398 also showed positive results in the DNA decatenation assay, these compounds might indeed induce DNA damage.

**Table 3:** IC_50_ values of selected artemisinin derivatives on the normal R6 and transformed R6 (R6T24) cells assessed by MTT assay.

**Compounds**	**3 days treatment (μM)**		**6 days treatment (μM)**	
	**R6**	**R6T24**	**R6**	**R6T24**
CMK-0298^a^^.^^1^	0.326	0.300	0.136	0.096
CMK-0398^a^^.^^1^	0.327	0.335	0.124	0.109
WWLL-013^b^^.^^2^	0.340	0.320	3.600	0.320
WWLL-022^c^^1^	0.175	0.180	0.093	0.089
WWLL-1098^b^^1^	0.865	0.861	0.222	0.178

a *Maximum test concentration: 50 μM*.

b *Maximum test concentration: 100 μM*.

c *Maximum test concentration: 25 μM*.

1 *DMSO as solvent*.

2 *tetrahydrofuran as solvent*.

As can be seen in Table 3, CMK-0298, CMK-0398, WWLL-013, WWLL-022, and WWLL-1098 were cytotoxic toward both cell lines in the low micromolar range. It is of interest to know that WWLL-013 showed a big discrepancy in IC_50_ between normal and the transformed R6 cells. This implies that this compound possess differential cytotoxicity against transformed cells, suggesting that there might be a leverage to use this compound to treat cancer with less harmful effect to the normal cells.

### Artemisinin does not directly intercalate into DNA to interfere with topo 1 activity

**Figure 7:**
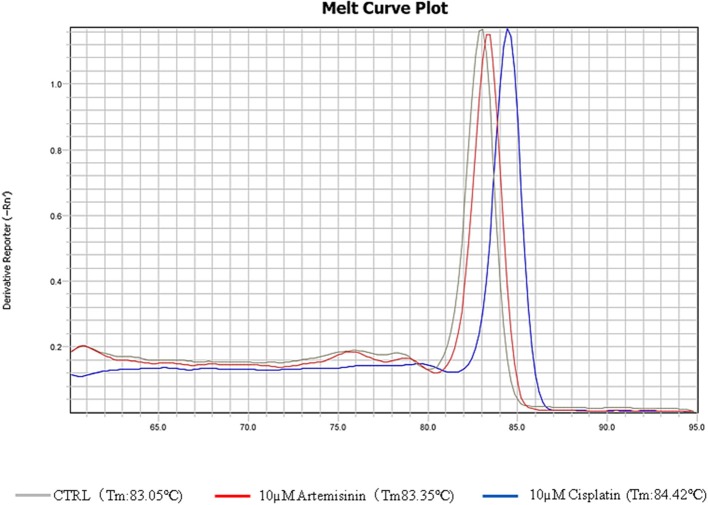
DNA melting curves of PCR products incubated in DMSO or test chemicals. The qPCR was conducted according to the “Materials and Methods.” DMSO, 10 μM cisplatin, or 10 μM artemisinin were added to the qPCR reaction.

In order to discriminate between DNA intercalation and Topo 1 inhibition, we conducted real time PCR assays for the house-keeping gene, actin and monitored its DNA dissociation curve and melting temperature in the presence of artemisinin or the known DNA intercalator and adduct-forming drug, cisplatin (Sangeetha Gowda et al., 2014). The melting temperature shifted from 80.05 to 84.42°C in the presence of cisplatin as determined by its DNA dissociation curve (Figure 7). On the other hand, the DNA dissociation curve in the presence of artemisinin was closely overlapping with that of the DMSO solvent control, suggesting that artemisinin did not exert DNA binding activity. This finding provides evidence that artemisinin acts directly on Topo 1 as inhibitor.

## Discussion

Molecular docking studies pointed to artemisinin derivatives as Topo 1 inhibitors. Interestingly, the selected artemisinin derivatives (CMK-0298, CMK-0398, WWLL 1098, WWLL-022, and WWLL-013) might be indeed Topo 1 inhibitors, since comparable binding energies and similar docking poses were observed with the known inhibitors. Further *in vitro* analyses supported their potential to be used as Topo 1 inhibitors.

WWLL-013 (−12.42 kcal/mol) and WWLL-022 (−12.21 kcal/mol) showed stronger binding than the known inhibitors on the wild-type Topo 1, whereas WWLL-1098 (−10.96 kcal/mol) and CMK-0398 (−10.72 kcal/mol) showed stronger binding than hydrolyzed topotecan (−10.53 kcal/mol) and camptothecin (−10.29 kcal/mol). CMK-0298 showed comparable binding energies (−9.97 kcal/mol) with the inhibitors. The selected compounds shared common interacting residues with the known inhibitors (Kollmannsberger et al., 1999; Baikar and Malpathak, 2010). WWLL-013, WWLL-022, and WWLL-1098 revealed six common residues, whereas CMK-0298 and CMK-0398 revealed two common residues. Molecular docking analysis on the N722S mutant Topo 1 revealed that camptothecin (−9.94 kcal/mol) docked to a different binding site, which is in corroboration with the hypothesis that the indicated mutation causes camptothecin resistance. The missing −NH_2_ group at serine 722 might be important for its binding. WWLL-013 (−12.58 kcal/mol) and WWLL-022 (−11.72 kcal/mol) showed comparable binding energies with topotecan (−12.66 kcal/mol) and four common interacting residues were observed with topotecan. CMK-0398 (−10.63 kcal/mol) and WWLL-1098 (–10.56 kcal/mol) showed stronger binding than hydrolyzed topotecan (−9.95 kcal/mol) and camptothecin (−9.94 kcal/mol), CMK-0298 showed comparable binding energy (–9.37 kcal/mol). The selected artemisinin derivatives showed strong interaction on wild-type and N722S mutant Topo 1 and shared common interacting residues, implying that they might be used as potent Topo 1 inhibitors. In order to prove the *in silico* findings, we performed experiments *in vitro*.

DNA decatenation assay (Sahai and Kaplan, 1986; Boos and Stopper, 2001) is a well-established method to detect DNA topoisomers, if an intercalator molecule is present. Under normal conditions, Topo 1 relaxes the closed circular DNA, and, hence, decreases the writhe to zero. In presence of an intercalator compound, the writhe will increase due to the decrease in the DNA twist leading to conformational changes and, thus, different bands at agarose gel. CMK-0298 and CMK-0398 indeed showed intercalator properties similar to bleomycin and ethidium bromide, implying that they possess DNA intercalator ability and thus Topo 1 inhibitory effect. Further evidence was provided by comet assay in order to evaluate DNA damage by the selected compounds. CMK-0298, CMK-0398, WWLL-013, and WLL-022 indeed induced DNA break. The fact that CMK-0298 and CMK-0398 also showed activity in decatenation assay, implies that they might be promising topoisomerase 1 inhibitors, as they induce DNA damage and possess intercalation activity.

Selected artemisinin derivatives showed cytotoxicity toward transformed rat fibroblast cells transfected with c-H-Ras oncogene in a low micromolar range. Moreover, the transformed cells were more sensitive than the normal cells to the all test derivatives, WWLL-013 in particular. This points out that they might possess anti-cancer activity as they are able to kill tumor model cells. Based on the DNA disassociation curve experiment (Figure 7), we showed that artemisinin does not directly intercalate into DNA to interfere with Topo 1 activity.

It has been recently reported that artemisinin and its derivatives such as dihydroartemisinin, artesunate, artemether, arteether possess a multi-target effect toward cancer as it influences various signaling pathways playing important roles in cancer progression (Li et al., 2016; Efferth, 2017). In the present study we identified artemisinin derivatives that could target Topo 1.

In conclusion, CMK-0298, CMK-0398, WWLL-013, WWLL-022, and WWLL-1098 possess anti-cancer activity and potential Topo 1 inhibitor properties as shown *in silico* and *in vitro*. Further preclinical and clinical studies are warranted in order to evaluate their therapeutic potential for clinical use.

## Author contributions

TE and WH conceived the study. OK performed the *in silico* experiments. VW, HF contributed to the *in silico* experiments. OK and TE wrote the manuscript. AC, ACLQ, VW performed the *in vitro* experiments. All the authors read the manuscript.

### Conflict of interest statement

The authors declare that the research was conducted in the absence of any commercial or financial relationships that could be construed as a potential conflict of interest.
