# Supplementary material for: Artemisinin Derivatives Target Topoisomerase 1 and Cause DNA Damage in Silico and in Vitro
Source: Front Pharmacol. 2017 Oct 9;8:711. doi: 10.3389/fphar.2017.00711 (PMC5640709; doi:10.3389/fphar.2017.00711)
Supplement: Supplementary file 1 [file Table1.DOCX]

**Supplementary materials:**

**Table S1- Chemical properties of the selected artemisinin derivatives**

| **Derivatives** | **Solvent** | **Maximum test conc. (uM)** |
| --- | --- | --- |
| **ARTEMISININ PARENT** | | |
| QINGHAOSU ^b,1^ | Ethanol | 100 |
| DIHYDROARTEMISININ ^a,1^ | Ethanol | 50 |
| ARTEMETHER ^b,1^ | Ethanol | 100 |
| AV-009 ^a,2^ | DMSO | 50 |
| AV-010 ^a,2^ | DMSO | 50 |
| WWLL-007 ^b,1^ | Ethanol | 100 |
| **11-AZA AND 2-DEOXY-11 AZAARTEMISININ DERIVATIVES** | | |
| AV-001 ^a,2^ | DMSO | 50 |
| AV-003 ^b,1^ | Ethanol | 100 |
| AV-007 ^a,2^ | DMSO | 50 |
| AV-008 ^a,2^ | DMSO | 50 |
| AV-004 ^a,2^ | DMSO | 50 |
| AV-006 ^a,2^ | DMSO | 50 |
| **10-ETHER AND 2-DEOXY-10-ETHER LINKED ARTEMISININ DERIVATIVES** | | |
| CAN-06 / CAN-25 PT A ^b,1^ | Ethanol | 100 |
| CAN-28 PT A ^b,2^ | DMSO | 100 |
| WWLL-021 ^b,2^ | DMSO | 100 |
| **WWLL-022 ^c,2^** | DMSO | 25 |
| WWLL-053 ^b,2^ | DMSO | 100 |
| D-CAN-25 PT A ^b,2^ | DMSO | 100 |
| **10-ESTER LINKED ARTEMISININ DERIVATIVES** | | |
| **WWLL-013 ^b,3^** | Tetrahydrofuran | 100 |
| WWLL-055 ^b,2^ | DMSO | 100 |
| WWLL-0998 ^b,2^ | DMSO | 100 |
| **WWLL-1098 ^b,2^** | DMSO | 100 |
| WWLL-1198 ^c,2^ | DMSO | 25 |
| ARTESUNATE ^b,1^ | Ethanol | 100 |
| **10-ARYL AND 2-DEOXY-10 ARYL LINKED ARTEMISININ DERIVATIVES** | | |
| WWLL-041 ^b,2^ | DMSO | 100 |
| WWLL-076 ^b,2^ | DMSO | 100 |
| **CMK-0298 ^a,2^** | DMSO | 50 |
| CMK-0498 ^b,2^ | DMSO | 100 |
| WWLL-0398 ^b,2^ | DMSO | 100 |
| WWLL-0498 ^a,2^ | DMSO | 50 |
| WWLL-0598 ^b,2^ | DMSO | 100 |
| WWLL-0698 ^b,2^ | DMSO | 100 |
| MW530 ^b,2^ | DMSO | 100 |
| D-WWLL-040 ^b,2^ | DMSO | 100 |
| D-WWLL-041 ^b,2^ | DMSO | 100 |
| **16-ALKYL LINKED ARTEMISININ DERIVATIVES** | | |
| AV-015 / WWLL-049 ^b,2^ | DMSO | 100 |
| WWLL-046 ^b,2^ | DMSO | 100 |
| **10-AMINOARYL ARTEMISININ DERIVATIVES** | | |
| WWLL-047 ^b,2^ | DMSO | 100 |
| WWLL-048 ^b,2^ | DMSO | 100 |
| WWLL-1598 ^b,2^ | DMSO | 100 |
| **10-AMINALKYL ARTEMISININ DERIVATIVES** | | |
| WWLL-071 ^b,2^ | DMSO | 100 |
| **CMK-0398 ^a,2^** | DMSO | 50 |
| **16 AMINO LINKED ARTEMISININ DERIVATIVES** | | |
| WWLL-044 ^b,2^ | DMSO | 100 |
| WWLL-045 ^b,2^ | DMSO | 100 |
